# A comparative study of catastrophic health expenditure in Zhejiang and Qinghai province, China

**DOI:** 10.1186/s12913-018-3658-9

**Published:** 2018-11-09

**Authors:** Xuemei Zhen, Hao Zhang, Xiaoqian Hu, Shuyan Gu, Yuanyuan Li, Yuxuan Gu, Minzhuo Huang, Xueshan Sun, Jingming Wei, Hengjin Dong

**Affiliations:** 1 Center for Health Policy Studies, School of Public Health, Zhejiang University School of Medicine, Hangzhou, Zhejiang China; 2 Hangzhou Normal University, Xuelin Street, Hangzhou, Zhejiang China

**Keywords:** Catastrophic health expenditure, Household, Zhejiang province, Qinghai province, China

## Abstract

**Background:**

China has made great achievements in health insurance coverage and healthcare financing; however, the rate of catastrophic health expenditure (CHE) was 13.0% in China in 2008, which is higher than that in some other countries. There remain some differences in life-style, national customs, medical conditions, and health consciousness in different provinces in China. This study aimed to compare the rates of households with CHE, further to explore the different performance of factors influencing CHE between Zhejiang and Qinghai province, China.

**Methods:**

Data were derived from the household surveys conducted in Zhejiang and Qinghai. Sampling on multi-stage stratified cluster random method was adopted. Household with CHE occurs when the out-of-pocket payment for health care equals to or exceeds 40% of a household’s income. Univariate and multivariate logistic regression analyses were used to identify the performance of factors of CHE.

**Results:**

A total of 1598 households were included in this study, including 995 in Zhejiang and 603 in Qinghai. The average rates of CHE in Zhejiang and Qinghai were 9.6 and 30.5%, respectively. We found that economic status of households and households headed by an employed person are the protective factors for CHE; and number of members with chronic diseases and number of inpatients in household are the risk factors for CHE in the two provinces. Besides, poor/low-insured households in Zhejiang; and households having outpatients and households headed by a minority person in Qinghai are more likely to experience the risk of CHE.

**Conclusions:**

This study highlights the importance of improving economic development, expanding employment, and adjusting policies to make greater efforts to protect chronic diseases patients, outpatients, and inpatients, further to reduce the risk of CHE. The Chinese government should pay more attention to the actual conditions in different provinces, further to make policy decisions according to the local knowledge.

## Background

The burden of health costs and the economic effect on households depend on the country’s health system and the ability of out-of-pocket (OOP) expenditure of households [1]. Protecting the population against the financial risks associated with ill health is one of the fundamental objectives of the health systems [2]. Globally, there is an average of about 32% of each country’s health expenditure from OOP payments [2]. It had estimated that approximately 150 million people globally are facing catastrophic expenditure annually because of high payments for health services [3]. Catastrophic health expenditure (CHE) is defined as OOP expenditure equaling to or exceeding 40% of household capacity to pay [4]. OOP expenditure and CHE are barriers to achieve universal health coverage [5], in which all people can obtain the health services they need without suffering financial hardship [6].

The health care reforms in China in the past aimed to establish a health system according with universal health coverage. There are three basic health insurances in China, including employer-based insurance, urban resident insurance, and new cooperative medical scheme. In recent years, many provinces have merge urban resident insurance and new cooperative medical scheme into urban and rural insurance [7]. Health insurance coverage has increased dramatically over the 20 years, from 30.2% in 1993 to 95.1% in 2013 [8]. The share of healthcare financing from OOP dropped from 59.97% in 2001 to 29.27% in 2015 [9]. However, the problems of expensive medical bills, difficult access to quality medical services, and poverty caused by diseases are still existing. The rate of CHE was 13.0% in China in 2008 [10], which is higher than some other countries [11–14]. Despite China’s great achievements in health insurance coverage and healthcare financing, it falls short up to universal health coverage in a more comprehensive sense [10].

China contains vast territories and abundant resources, there remain some differences in life-style, national customs, medical conditions, and health consciousness. In addition, the processes of health care reform are wildly different in different provinces. It is necessary to provide targeted and practical suggestions based on the timely regional data.

As an important part of healthcare financing, a lot of studies focus on CHE. Most studies have measured the distribution and determinants of CHE in China [10, 14] and in other countries [11–13]. Some studies have explored CHE in one of provinces in China [15, 16]. However, to our knowledge, very few studies have compared the occurrence and the performance of factors influencing CHE in two different provinces, China.

In this study, first objective is to quantify the extent of households faced CHE between Zhejiang and Qinghai, China. Secondly, it is aimed to identify the different performance of characteristics that affect households to incur health catastrophe in the two provinces. Thus, in designed the health systems, policy makers can make decisions according to the local knowledge that whether any characteristics are more vulnerable to CHE.

## Methods

### Data source and sampling

The data used to calculate CHE were acquired from two household surveys conducted in Zhejiang in 2015 and Qinghai in 2016, which represent the eastern developed area and the western developing area, China.

Zhejiang, which covers 105,500 km^2^, is located in south-east coastal area of China, and had a population of about 5,539,000 and a Gross Domestic Product (GDP) of about the United States (US) $ 1.23 trillion in 2015 [9, 17, 18]. It is an economically developed region in China. Since 2012, Zhejiang has steadily carried out the mechanism of “Double Sinking and Two Lifting” and sign contracts with general practitioners, deepened the reform of medical and health system, set up hierarchical medical system, and improved the ability of primary health care service. Qinghai, which covers 721,000 km^2^, is located in the west of China, and had a population of about 588,000 and a GDP of about US $ 0.60 trillion in 2015 [9, 18, 19]. It is economically underdeveloped region in China. The minority nationalities accounted for 47.71% of the population in Qinghai, which has geographical characteristics and distinctive national features. Qinghai is enforce to implement initial diagnosis at primary health care institutions and two-way referral system, which closely combined between hierarchical medical system and the reform of medical insurance payment system [20]. Both Zhejiang and Qinghai are pilot provinces in overall health care reform nationwide [21], who play the important roles in health care reform and “healthy China”.

Sampling on multi-stage stratified cluster random method was adopted in this study [15]. According to cluster analysis from the perspective of GDP, developed and underdeveloped areas in Zhejiang and Qinghai were randomly selected, and we added relatively developed areas in Qinghai due to the huge gap between the rich and the poor [22]. In the first stage, we randomly selected Jiashan county in jiaxing city and Jinyun county in Lishui city to represent developed areas and underdeveloped areas in Zhejiang. We randomly selected Chengxi district in Xining city, Pingan district and Huzhu county in Haidong city, and Jianzha county in Huangnan Tibetan autonomous prefecture to represent developed, relatively developed, and underdeveloped areas in Qinghai. In the second stage, we randomly selected one urban or one rural area from each county or district to represent street or village. In the third stage, based on the proportion of permanent residents, a number of households from each street or village were randomly sampled. Finally, there were two households with missing data and 19 households without health expenditures; thus, we exclude them, making the final sample size 1598.

### Data collection and quality control

According to the questionnaire in the Fifth National Health Services Survey, which has been shown to be consistent and reliable [10], we designed the questionnaire in this survey [8], including six parts: general household information, individual information, disease information in the last two weeks of survey, hospitalization information in the last one year of survey, patients’ satisfaction and accessibility in basic medical facilities, and willingness of getting medical treatment. Face-to-face household interviews were conducted by qualified investigators with the great help of local health-related governments. Professors and postgraduate students in Zhejiang University were responsible for the strict quality control.

### Indicators and data analyses

In this study, the main indicator is CHE, which occurs when the OOP payment for health care equals to or exceeds 40% of a household’s income [15], which is related to capacity to pay of household, healthcare demand of household, and baseline characteristics of household head. Actually, some previous studies used household consumption expenditure rather than household income to calculate the rate of CHE. However, Chinese residents’ saving rate is as high as 51.8% [15]. In other words, household income is greater than household consumption expenditure in most Chinese households. The capacity to pay would be underestimated and the rate of CHE would be overestimated, if household consumption expenditure were used to measure capacity to pay. Therefore, we used household income to measure household’s capacity to pay in this study. There are two different ways about household income: disposable income of urban households and net income of rural households.

This study uses only a portion of the data gathered by the questionnaire. Based on the variables in the questionnaire and the main indicator in our study, we defined some new variables about household that may influence CHE. These included capacity to pay of household: household size, which is the number of permanent residents per household, household income per year, household consumption expenditure per year, household health expenditure per year, poor/low-insured household, which is the poor or low-insured household identified by the local government, proportion of members with health insurance in household; healthcare demand of household: number of members over 60 years in household, number of members with chronic diseases in household in the last six months, number of outpatients in household in the last two weeks of the survey, and number of inpatients in household in the last one year of the survey; and baseline characteristics of household head: residency, sex, nationality, marital status, which is marital status in the marriage law, educational status, and employment status.

First, we compared the characteristics of households and households with CHE between Zhejiang and Qinghai. χ^2^ or Fisher’s exact test was used for categorical variables, and T-test or Manny-Whitney test was for continuous variables. Then, univariate logistic regression analyses were used to describe the effect of each factor on CHE and stepwise multivariate logistic regressions to explore the effect of factors on CHE. In the regression analyses, CHE was taken as the dependent variable, characteristics of households with CHE were taken as the explanatory variables. We assumed that all above variables have a positive association with CHE, except household size, household income per year, and proportion of members with health insurance in household, and household headed by a man, a married person, or an employed person.

All expenses in our analysis were converted to 2015 US $ values, using the consumer price index of China and the 2015 purchasing power parities [23, 24]. All tests were two-sided and a *p*-value< 0.05 was deemed to indicate statistical significance. SPSS 21.0 was used for all statistical analyses.

## Results

### Household characteristics

A total of 1598 households were included in this study, including 995 in Zhejiang and 603 in Qinghai. Of these, the average rates of CHE in the two provinces, Zhejiang, and Qinghai were 17.5, 9.6, and 30.5%, respectively.

Compared with households in Qinghai, households in Zhejiang were significantly associated with higher household income, and higher household consumption expenditure, but lower household health expenditure, and smaller household size and poor/low-insured household. Household heads in Zhejiang were more likely to be rural, be male, or be married status, but less likely to be minority or be illiteracy than those in Qinghai. On average, households in Qinghai were significantly associated with more members with chronic diseases in household and more inpatients in household, but fewer members over 60 years in household than those in Zhejiang. There were no significant differences between the two provinces in the aspects of proportion of members with health insurance in household and number of outpatients in household (Table 1).

**Table 1:** Household characteristics in Zhejiang and Qinghai province, China

Household characteristics	Households				Households with CHE			
	Total (*n* = 1598)	Zhejiang (*n* = 995)	Qinghai (*n* = 603)	P-value	Total (*n* = 280)	Zhejiang (*n* = 96)	Qinghai (*n* = 184)	*P*-value
Household with CHE, *n* (%)	280 (17.5)	96 (9.6)	184 (30.5)	< 0.000				
Household income per year, mean (SE) (US $)	17,430.06 (459.00)	21,539.81 (539.19)	10,648.63 (752.56)	< 0.000	6507.53 (528.52)	9462.44 (1187.59)	4965.83 (477.67)	0.001
Household consumption expenditure per year, mean (SE) (US $)	10,934.17 (244.05)	12,638.74 (283.19)	8121.48 (423.20)	< 0.000	8252.97 (772.65)	10,073.46 (1168.00)	7303.15 (1000.57)	0.021
Household health expenditure per year, mean (SE) (US $)	2204.33 (121.62)	2013.31 (110.52)	2519.53 (265.40)	0.002	5774.66 (606.82)	6912.54 (798.13)	5180.98 (822.29)	0.022
Household size, mean (SE)	3.04 (0.03)	2.86 (0.04)	3.35 (0.06)	< 0.000	3.19 (0.09)	2.53 (0.11)	3.54 (0.11)	< 0.000
Poor/low-insured household, n (%)	91 (5.7)	25 (2.5)	66 (10.9)	< 0.000	41 (14.6)	13 (13.5)	28 (15.2)	0.707
Rural household head, n (%)	859 (53.8)	583 (58.6)	276 (45.8)	< 0.000	181 (64.6)	71 (74.0)	110 (59.8)	0.019
Male household head, n (%)	1202 (75.2)	770 (77.4)	432 (71.6)	0.010	223 (79.6)	76 (79.2)	147 (79.9)	0.886
Minority household head, n (%)	171 (10.7)	14 (1.4)	157 (26.0)	< 0.000	80 (28.6)	3 (3.1)	77 (41.8)	< 0.000
Married household head, n (%)	1334 (83.5)	859 (86.3)	475 (78.8)	< 0.000	222 (79.3)	76 (79.2)	146 (79.3)	0.972
Educational status of household head, n (%)				< 0.000				0.001
Illiteracy	166 (10.4)	56 (5.6)	110 (18.2)		68 (24.3)	10 (10.4)	58 (31.5)	
Primary school	448 (28.0)	305 (30.7)	143 (23.7)		99 (35.4)	43 (44.8)	56 (30.4)	
Secondary school	516 (32.3)	334 (33.6)	182 (30.2)		71 (25.4)	29 (30.2)	42 (22.8)	
High/technical school or above	468 (29.3)	300 (30.2)	168 (27.9)		42 (15.0)	14 (14.6)	28 (15.2)	
Employment status of household head, n (%)				< 0.000				0.001
Employed	1027 (64.3)	673 (67.6)	354 (58.7)		134 (47.9)	38 (39.6)	96 (52.2)	
Retired	305 (19.1)	192 (19.31)	113 (18.7)		61 (21.8)	22 (22.9)	39 (21.2)	
Unemployed	266 (16.6)	130 (13.1)	136 (22.6)		85 (30.4)	36 (37.5)	49 (26.6)	
Proportion of members with health insurance in household (%),mean (SE)	97.04 (0.32)	96.95 (0.39)	97.20 (0.55)	0.164	97.73 (0.67)	98.75 (0.66)	97.20 (0.97)	0.502
Number of members over 60 years in household, mean (SE)	0.70 (0.02)	0.78 (0.03)	0.56 (0.03)	< 0.000	0.86 (0.05)	1.14 (0.09)	0.72 (0.06)	< 0.000
Number of members with chronic diseases in household in the last six months, mean (SE)	0.39 (0.02)	0.33 (0.02)	0.48 (0.03)	< 0.000	0.74 (0.05)	0.74 (0.08)	0.74 (0.06)	0.963
Number of outpatients in household in the last two weeks of the survey, mean (SE)	0.18 (0.01)	0.17 (0.01)	0.19 (0.02)	0.486	0.39 (0.04)	0.45 (0.07)	0.35 (0.04)	0.225
Number of inpatients in household in the last one year of the survey, mean (SE)	0.24 (0.1)	0.17 (0.01)	0.34 (0.02)	< 0.000	0.63 (0.04)	0.63 (0.07)	0.63 (0.05)	0.967

*CHE* catastrophic health expenditure; *SE* standard error; interquartile range; *US* United States

Household size: number of permanent residents in household; Poor/low-insured household: poor or low-insured household identified by the local government; Married: married status in the marriage law

χ^2^ or Fisher’s exact test was used for categorical variables, and T-test or Manny-Whitney test was for continuous variables

### Household characteristics in relation to catastrophic health expenditure

Compared with households with CHE in Qinghai, those in Zhejiang were significantly associated with higher household income, higher household consumption expenditure, higher household health expenditure, and more members over 60 years in household, but smaller household size. Households headed by a rural person in Zhejiang; and households headed by a minority person or an illiteracy in Qinghai were more likely to face CHE. There were no significant differences between the two provinces in the terms of poor/low-insured household, sex and married status of household head, proportion of members with health insurance, number of members with chronic diseases, outpatients and inpatients in household (Table 1).

### Factors influencing catastrophic health expenditure in univariate analysis

In Zhejiang, all household characteristics were significantly associated with CHE, except three variables of sex, nationality of household head, and proportion of members with health insurance in household. Household income, household size, household headed by a married person or an employed/retired person were protective factors for CHE. In Qinghai, all household characteristics were significantly associated with CHE, except four variables of marital status, educational status (secondary school vs high/technical school or above), employment status of household head, and proportion of member of health insurance in housheold. Household income per year is the only protective factor for CHE (Table 2).

**Table 2:** Factors influencing catastrophic health expenditure in univariate logistic regression model

Variables	Zhejiang				Qinghai			
	b	S.E.	OR	*P*-value	b	S.E.	OR	*P*-value
Household income per year	−0.0001	0.00001	1.000	< 0.000	−0.0002	0.00002	1.000	< 0.000
Household size	−0.263	0.097	0.768	0.007	0.132	0.063	1.141	0.035
Poor/low-insured household	2.449	0.416	11.577	< 0.000	0.588	0.267	1.800	0.028
Rural residency of household head	0.764	0.242	2.147	0.002	0.818	0.181	2.266	< 0.000
Male household head	0.116	0.264	1.122	0.661	0.625	0.212	1.868	0.003
Minority nationalities of household head	0.957	0.660	2.604	0.147	1.115	0.194	3.049	< 0.000
Married household head	−0.575	0.270	0.563	0.034	0.050	0.218	1.051	0.819
Educational status of household head (reference: high/technical school or above)								
Illiteracy	1.491	0.443	4.441	0.001	1.719	0.282	5.577	< 0.000
Primary school	1.210	0.319	3.353	< 0.000	1.169	0.269	3.218	< 0.000
Secondary school	0.664	0.336	1.942	0.048	0.405	0.272	1.500	0.136
Employment status of household head (reference: Unemployed)								
Employed	−1.856	0.258	0.156	< 0.000	−0.415	0.215	0.661	0.054
Retired	−1.085	0.300	0.338	< 0.000	−0.066	0.267	0.936	0.803
Proportion of members with health insurance in household	0.021	0.015	1.022	0.146	0.00005	0.007	1.000	0.994
Number of members over 60 years in household	0.448	0.113	1.565	< 0.000	0.346	0.108	1.414	0.001
Number of members with chronic diseases in household in the last six months	0.907	0.141	2.476	< 0.000	0.749	0.127	2.115	< 0.000
Number of outpatients in household in the last two weeks of the survey	1.086	0.181	2.962	< 0.000	1.016	0.191	2.763	< 0.000
Number of inpatients in household in the last one year of the survey	2.150	0.230	8.588	< 0.000	1.654	0.195	5.227	< 0.000

*SE* standard error; *OR* odds ratio

Household size: number of permanent residents in household; Poor/low-insured household: poor or low-insured household identified by the local government; Married: married status in the marriage law

### Factors influencing catastrophic health expenditure in multivariate analysis

We found that the independent factors for households with CHE in both Zhejiang and Qinghai are household income, employed status of household head, number of members with chronic diseases in household, and number of inpatients in household; and the first two variables had negative effects on CHE. In addition, other independent factors for CHE in Zhejiang included poor/low-insured household; for CHE in Qinghai were household headed by a minority person, and number of outpatients in household. The most important independent factor for CHE in the two provinces was number of inpatients in household, followed by poor/low-insured household in Zhejiang and minority household head in Qinghai (Table 3).

**Table 3:** Factors influencing catastrophic health expenditure in multivariate logistic regression model

Variables	Zhejiang				Qinghai			
	b	S.E.	OR	*P*-value	b	S.E.	OR	*P*-value
Household income per year	−0.0001	0.00002	1.000	< 0.000	−0.0002	0.00002	1.000	< 0.000
Poor/low-insured household	1.797	.507	6.029	< 0.000				
Minority household head					0.724	0.233	2.063	0.002
Employment status of household head (reference: unemployed)								
Employed	−0.710	0.313	0.492	0.023	−0.652	0.257	0.521	0.011
Retired	−0.149	0.394	0.861	0.704	−0.180	0.325	0.835	0.580
Number of members with chronic diseases in household in the last six months	0.528	0.182	1.695	0.004	0.452	0.161	1.572	0.005
Number of outpatients in household in the last two weeks of the survey					0.505	0.231	1.657	0.029
Number of inpatients in household in the last one year of the survey	1.752	0.232	5.764	< 0.000	0.859	0.186	2.361	< 0.000
(Constant)	−1.007	0.282	0.365	< 0.000	−0.133	0.276	0.876	0.631
Model	< 0.000				< 0.000			
−2 log likelihood	409.504				550.674			
Nagelkerke R^2^	0.415				0.384			
Cox & Snell R^2^	0.200				0.272			

*SE* standard error; *OR* odds ratio

Household size: number of permanent residents in household; Poor/low-insured household: poor or low-insured household identified by the local government; Married: married status in the marriage law

## Discussion

To the best of our knowledge, it is the first study to analyze the distribution and performance of factors of CHE in Zhejiang and Qinghai. It is also the first study to compare the differences of households with CHE in the two provinces, China.

CHE reflects the economic burden of households and the financial barriers to receive health care. In our study, households in Qinghai are at higher risk of experiencing CHE than households in Zhejiang. It may be closely related to regional economic development and healthcare demand in the two provinces. Total health expenditure in Zhejiang of about US $ 65 billion was over ten times as many as those in Qinghai (US $ 6 billion), total health expenditure per capita in Zhejiang was US $ 113 higher than that in Qinghai, and household income in Zhejiang is two times higher than that in Qinghai [9]. Although total health expenditure account for GDP in Zhejiang was lower than that in Qinghai (5.25% vs 8.93%); and OOP payment as a share of total health expenditure in Zhejiang was relatively higher than that in Qinghai in 2015 (29.4% vs 23.90%), they did not affect the higher rate of CHE in Qinghai [9] because of Zhejiang’s GDP is twice that of Qinghai [18].

Low-income households and households headed by an unemployed person are more likely to suffer CHE, which are consistent with the findings in other studies [10, 12, 14, 25]. Therefore, strategies that increase income among low-income households and narrow the income gap can be implemented, or efforts that improve the employment ability and brighten the employment environment should be made, further to reduce the effects of economic status or employment status for CHE, respectively [14].

The risk of CHE increased when the members in household in Qinghai went to hospital for outpatient or inpatient services. However, in Zhejiang, only inpatient services did affect the risk of CHE. It is reported that households with one or more inpatients or outpatients are at higher risks to encounter CHE because of the higher demands of health care [26]. However, residents in developing regions who get access to outpatient service are at higher risk of experiencing CHE than those in developed regions. Therefore, policies for outpatients or inpatients should be adjusted according to the local conditions, for example, great efforts should be made to vigorously develop insurance for catastrophic illness in the two provinces, and adjust reimbursement ratio for outpatients in Qinghai, in order to reduce the rate of “poverty caused by diseases” [14].

Poor/low-insured households are at higher risk of experiencing CHE for households in Zhejiang, which is similar to other studies [14, 27, 28]. It is necessary to give greater government support for poor/low-insured households, especially in Zhejiang. In addition, households headed by a minority person are significantly associated with higher risk for CHE in Qinghai but not in Zhejiang. The minority nationalities accounted for about half of the population in Qinghai [19], who lives in hard-to-reach regions with abominable natural conditions, and works on agricultural and animal husbandry production. Besides, historical and social factors also restrict economy development among minorities in Qinghai [29]. According to the results in Chinese General Social Survey, household income of Han nationality was US $ 19,400, US $ 12,892 higher than that of minority nationalities; however, minority nationalities were associated with higher OOP for health care than Han nationality in China in 2014 [30]. It is reported that rapid population growth, and early marriage and early pregnancy are common in Qinghai, especially minority areas [29].

We found that health insurance did not significantly affect CHE in both univariate and multivariate analyses, which is similar to some studies [15, 31]. This demonstrates that health insurance, which mainly include three basic health insurances in China, actually have not reduced the risk of CHE. The weak performance of basic health insurance maybe related with the fact that almost residents in China are covered by basic health insurance [9]. Therefore, it is necessary to redesign basic health insurance or increase demand for commercial insurance in order to protect the households against CHE.

Some limitations of our study are worth noting. First, the data in our analysis is based on the remembrance of expenses in different time periods, which was taken to a weekly, monthly, or yearly unit for analysis, the actual prevalence of outpatients, inpatients and chronic diseases is probably higher than that reported in China. In addition, these results may not directly translate to other provinces in China; thus, future relevant studies in other provinces are needed.

## Conclusion

The findings revealed that there are tremendous differences in the rates of CHE in Zhejiang and Qinghai. Economic status of households and households headed by an employed person are major protective factors for CHE; and number of members with chronic diseases and number of inpatients in household are the risk factors for CHE in the two provinces. Besides, poor/low-insured household in Zhejiang; and households having outpatients and households headed by a minority person in Qinghai are more likely to experience the risk of CHE. Therefore, it is necessary to improve economic development, expand employment, and adjust policies to make greater efforts to protect chronic diseases patients, outpatients, and inpatients, further to reduce the risk of CHE. The Chinese government should pay more attention to the actual conditions in different provinces, further to make policy decisions according to the local knowledge.

## Abbreviations

CHE: Catastrophic health expenditure
GDP: Gross domestic product
OOP: Out-of-pocket
US: United States
